# *Annona muricata* L. Leaf Methanolic Extract Modulates Glucose Homeostasis and Improves Pancreatic Hormones Protein Expression in Diabetic Rats

**DOI:** 10.3390/life16081219

**Published:** 2026-07-23

**Authors:** Luis Rodrigo Morales-Arzate, Alejandra Hernández-Fonseca, Juan Carlos Pizano-Andrade, José Alfredo Domínguez-Rosales, Zamira Helena Hernández-Nazará, Efigenia Montalvo-González, Carmen Magdalena Gurrola-Díaz, Bertha Ruíz-Madrigal, Montserrat Maldonado-González, Belinda Vargas-Guerrero

**Affiliations:** 1 Instituto de Investigación en Enfermedades Crónico Degenerativas (IECD), Departamento de Biología Molecular y Genómica, Centro Universitario de Ciencias de la Salud, Universidad de Guadalajara, Guadalajara 44340, Mexico; luisrmoralesarzate@gmail.com (L.R.M.-A.); alejandrahdzfonseca@gmail.com (A.H.-F.); jose.drosales@academicos.udg.mx (J.A.D.-R.); zamira.hernandez@academicos.udg.mx (Z.H.H.-N.); carmen.gurrola@academicos.udg.mx (C.M.G.-D.); 2 Departamento de Botánica y Zoología, Centro Universitario de Ciencias Biológicas y Agropecuarias, Universidad de Guadalajara, Camino Ramón Padilla Sánchez 2100, Las agujas, Zapopan 44600, Mexico; pizanjc@gmail.com; 3 Laboratorio Integral de Investigación en Alimentos, División de Estudios de Posgrado, Instituto Tecnológico de Tepic, Av. Tecnológico 2595, Tepic 63175, Mexico; emontalvo@ittepic.edu.mx; 4 Centro de Investigación en Enfermedades Infectocontagiosas (CIEIC), Departamento de Microbiología y Patología, Centro Universitario de Ciencias de la Salud, Universidad de Guadalajara, Guadalajara 44340, Mexico; bertha.ruiz@academicos.udg.mx (B.R.-M.); montserrat.maldonado@academicos.udg.mx (M.M.-G.)

**Keywords:** *Annona muricata*, diabetes, leptin, insulin, glucagon

## Abstract

Type 2 diabetes mellitus represents an important worldwide health problem. *Annona muricata* L. (AM) is used for diabetes management. Additional research is required to elucidate its hypoglycemic mechanism; we evaluate its effect on serum leptin, glucagon, insulin, neuropeptide Y concentrations and protein levels of glucagon and insulin in diabetic model. Diabetes was induced in male Wistar rats using a high-fat diet (HFD) combined with 35 mg/kg streptozotocin (STZ). The animals were randomly assigned to four groups (*n* = 5): Ctrl (healthy animal), HFD (untreated HFD), AM (50 mg/kg of AM) and HFD Met (300 mg/kg of metformin). Treatments were administered orally for four weeks. Serum levels of leptin, glucagon, NPY, insulin, lipid renal and hepatic profiles were quantified before and after treatment. Pancreatic insulin and glucagon content were assessed by immunohistochemistry. Two-way ANOVA with repeated measures (intragroup) and ANOVA (intergroup) were used. The HFD AM group showed lower serum glucose (41.2%), total cholesterol (38.8%), triglycerides (69.6%) and NPY (73.4%) levels than pretreatment values (*p* < 0.05). Increased serum leptin concentration (53.7%), pancreatic insulin (42.0%), and glucagon (48.0%) protein levels (*p* < 0.05) were observed. Our study provides additional insights into the hypoglycemic effects of AM and supports its potential use as a complementary treatment for diabetes.

## 1. Introduction

Type 2 diabetes mellitus (T2DM) is a chronic metabolic disorder characterized by hyperglycemia due to impaired insulin action. The main causes associated with the development of T2DM include age, inadequate dietary habits, such as the consumption of ultra-processed foods, physical inactivity, overweight, and obesity, and various genetic factors that have not yet been clearly defined [1]. Despite the use of several drugs for diabetes management, metformin remains the standard first-line drug used to treat T2DM. It decreases both intestinal glucose absorption and hepatic glucose production and improves muscle insulin sensitivity, among other effects [2,3]. Therefore, in this study, metformin was used as a control. Recently, interest has increased in investigating foods and bioactive compounds that help control hyperglycemia in diabetes. In this context, *Annona muricata* L. (AM) has been reported to exert a hypoglycemic effect by modulating various metabolic pathways. This tropical fruit plant belongs to the Annonaceae family, is commonly known as soursop or graviola, and is cultivated in warm regions of the Americas, Africa, and Asia. This species is recognized for its therapeutic properties, which are attributed to more than 200 bioactive compounds. Due to its pharmacological effects, it has been used in traditional medicine for the treatment of conditions such as hypertension, cancer, bacterial diseases, and diabetes [4,5,6].

Different parts of the plant, such as the stem, pulp, and leaves, have traditionally been used for the treatment of diabetes. It has been reported that methanolic and aqueous extracts of AM leaves reduce blood glucose levels in streptozotocin (STZ)-induced diabetic rats [4]. In addition, in a study evaluating different orally administered doses of an aqueous extract of AM leaves in C57BL/6 mice with diet-induced obesity, a reduction in LDL and VLDL cholesterol concentrations, triglyceride levels, and the atherogenic index was reported [5]. Likewise, another study showed that, when compared with a diabetic control group, administering a methanolic extract of AM leaves reduced fasting blood glucose, glycated hemoglobin (HbA1c), and homeostatic model assessment of insulin resistance (HOMA-IR), while increasing glucose transporter 2 (GLUT2) and phosphorylated AKT (AKTp) levels and decreasing the protein levels of fatty acid synthase (FAS), sterol regulatory element-binding protein 1c (SREBP1c), CCAAT/enhancer-binding protein alpha (C/EBPα), and peroxisome proliferator-activated receptor gamma (PPARγ) [6,7].

Furthermore, inhibition of α-amylase, α-glucosidase, and angiotensin-converting enzyme-1 activity has been reported in an in vitro study using aqueous extracts of AM pulp, pericarp, and seeds [8]. AM has polyphenols that differ in quantity and composition between the different parts of the plant, such as pulp, pericarp, seeds, and leaves. It is considered that these might be involved in glycemic control [9,10]. However, it is important to clarify the complete mechanism of action to assess its potential use as a complementary treatment for T2DM. Therefore, we aimed to evaluate the effect of a methanolic extract of AM on serum leptin, glucagon, neuropeptide Y (NPY), and insulin levels, as well as on the pancreatic protein expression of glucagon and insulin in diabetic rats.

## 2. Materials and Methods

### 2.1. Materials

Casein high nitrogen (80 mesh) (Dyets, Inc., Bethlehem, PA, USA), AIN-93-VX vitamin mix (Dyets, Inc., Bethlehem, PA, USA), AIN-93G-MX mineral mix (Dyets, Inc., Bethlehem, PA, USA), sodium choleate (Sigma Aldrich, St. Louis, MO, USA), Cholesterol Nat’l Formulary (Dyets, Inc., Bethlehem, PA, USA), rodent lab chow diet 5001 (Labdiet, St. Louis, MO, USA), tiletamine–zolazepam (Zoletil^®^50, Virbac, Carros, Francia), glucose (BioSystems, Barcelona, Spain), aspartate aminotransferase (BioSystems, Barcelona, Spain), urea (BioSystems, Barcelona, Spain), creatinine (BioSystems, Barcelona, Spain), total cholesterol (BioSystems, Barcelona, Spain), triglycerides (BioSystems, Barcelona, Spain), streptozotocin (Sigma Aldrich, St. Louis, MO, USA), methanol, (Sigma Aldrich, St. Louis, MO, USA), insulin rat ELISA kit (DRG International, Inc., Springfield, NJ, USA), rat leptin ELISA kit (Abcam, Cambridge, UK), rat neuropeptide Y ELISA kit (MyBiosource, Inc., San Diego, CA, USA), Quantikine ELISA kit (R&D Systems, Inc., Minneapolis, MN, USA), insulin (C27C9) rabbit monoclonal antibody (Cell Signaling Technology, Danvers, MA, USA), anti-glucagon antibody, ab92517, (Abcam, Cambridge, UK), Mouse/Rabbit ImmunoDetector HRP/DAB Detection System (BIO SB, Santa Barbara, CA, USA).

### 2.2. Methods

**Figure 1:**
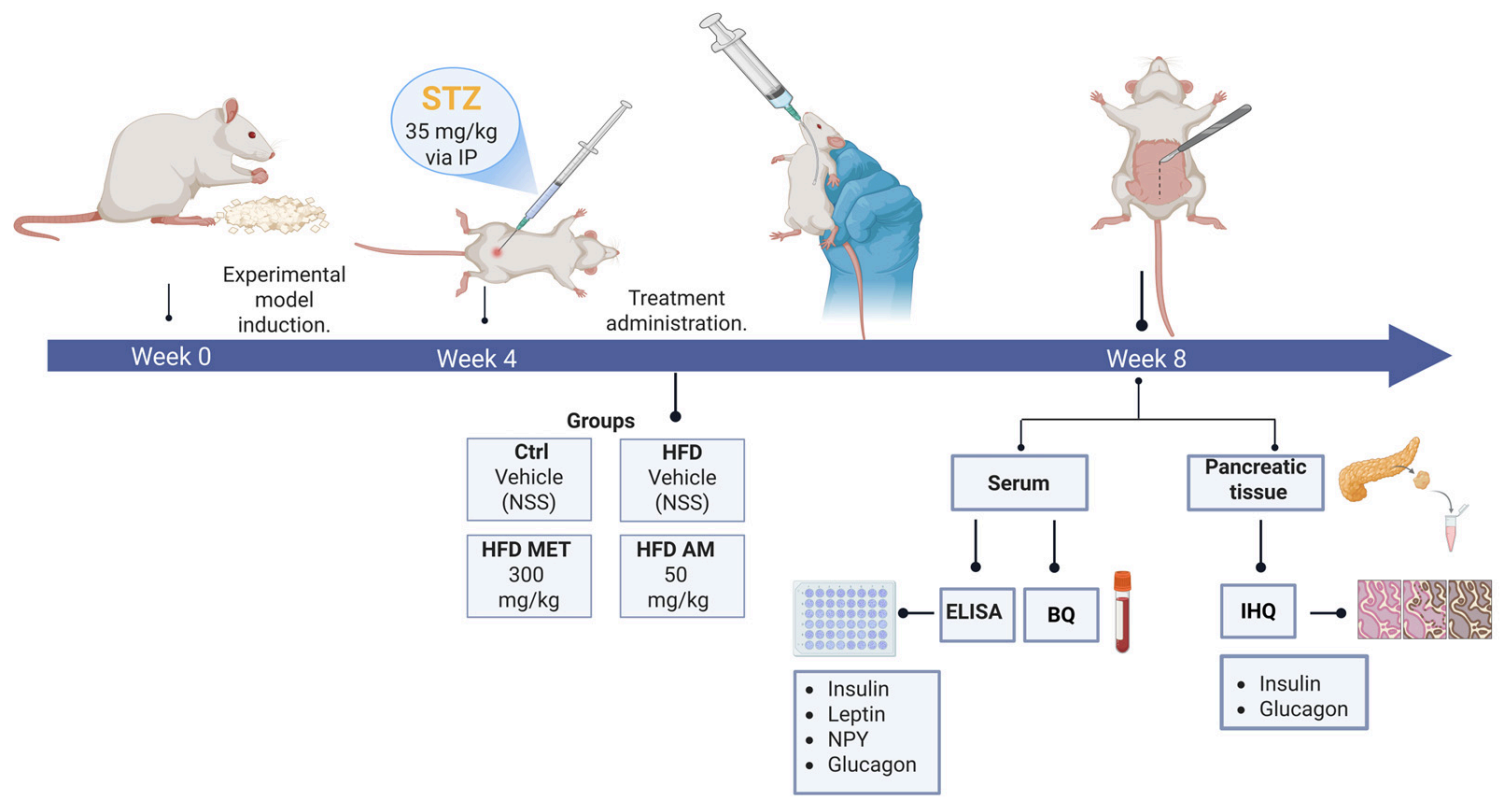
Outline of the methodology used. Illustration created in BioRender. Vargas Guerrero, B. (2026) https://BioRender.com/1wlq58m. Licensed under CC BY 4.0.

The methods employed are detailed below and summarized in Figure 1.

#### 2.2.1. Plant Material Collection

AM leaves were collected in the municipality of Tecomán, Colima, Mexico (18°58′36.3″ N 103°51′24.2″ W). A plant specimen was identified at the National Laboratory for Plant Identification and Characterization (LaniVeg), Universidad de Guadalajara (voucher specimen SIST-TRA-2019-04), and a reference specimen was deposited in the Luz María Villarreal de Puga Herbarium of the Institute of Botany, Universidad de Guadalajara. The identified leaves were dried in an oven at 50 °C for 48 h and then ground into a fine powder and stored until use.

#### 2.2.2. Ultrasound-Assisted Methanolic Extraction

For the methanolic extraction of AM leaves, 0.4 g of dried powdered leaves was weighed and mixed with 400 mL of 80% methanol. The mixture was placed in an ultra sonic bath for 20 min at room temperature, 70 W, and 40 kHz (Ultrasonic cleaner, catalog JP-040S, Skymen, Bao’an, Shenzhen, China). Subsequently, the samples were filtered and concentrated using a rotary evaporator (Buchi R-215, Flawil, Suiza)) at 50 °C until complete methanol evaporation. To accurately weigh the extract and ensure the administration of precise doses, it was lyophilized using a FreeZone 4.5 freeze dryer (Labconco, Kansas, MO, USA), and the sample was stored at 4 °C until use. This procedure was carried out ten times to obtain enough AM extract. The powder obtained from the lyophilization of each extraction was uniformly mixed to obtain a single lyophilized extract, which was subsequently used for the HPLC analysis and treatment of the HFD AM group.

#### 2.2.3. Characterization of the Methanolic Extract by HPLC

A total of 0.5 g of lyophilized AM extract was weighed and mixed with 5 mL of an acidified methanolic solution. The mixture was extracted with an orbital shaker for 1 h and centrifuged at 13,000 rpm for 10 min at 4 °C (Hermle Labortechnik, Wehingen, Germany, model Z36HK) to obtain the supernatant. Five milliliters of acetone was added to the remaining residue, followed by agitation, centrifugation, and collection of the supernatant. The combined extracts were evaporated to dryness using cold air. The dried sample was resuspended in 1.5 mL of HPLC-grade methanol. Phenolic compounds were identified using HPLC, following the method of Nolasco-González et al. [11]. Extracts (10 μL) were injected into an HPLC system (Agilent Technologies 1260 Infinity, Waldbronn, Alemania) equipped with a photodiode array detector and a reverse-phase Poroshell 120 EC-C18 column (particle size 2.7 μm, 4.6 mm internal diameter, 100 mm length; Agilent Technologies). The mobile phase consisted of acidified water with 0.10% trifluoroacetic acid (eluent A) and acetonitrile (eluent B). Standards and extracts were analyzed using the following gradient program: 0% B; 0–10 min, 10% B; 10–15 min, 20% B; 15–20 min, 25% B; 20–35 min, 35% B; 35–55 min, 75% B; 55–57 min, 100% B; 57–62 min, 65% B; 62–65 min, 35% B; and 65–70 min, 0% B, at a flow rate of 0.5 mL/min. Phenolic compounds were detected at 270–320 nm. Phenolic quantification was performed using 21 standards and their characteristics are described in Supplementary Table S1.

#### 2.2.4. Experimental Groups

The sample size was determined using statistical power analysis and the resource equation method for animal experiments, resulting in five rats per experimental group [12]. Then, twenty male Wistar rats weighing 150–200 g were obtained from the bioterium of Centro Universitario de Ciencias de la Salud, Universidad de Guadalajara. Experimental animals were housed 3 per cage, under standard conditions with a 12 h light/dark cycle, 55 ± 5% humidity, and a temperature of 24 ± 2 °C, with free access to food and water (ad libitum). Additionally, the cages were provided for physical environmental enrichment. To reduce the possible confounding factors, the animals were randomly allocated to study groups using a random number generator; environmental conditions were standardized as mentioned; all animals were managed in the same manner; and they were maintained isolated from other species. The cages were distributed horizontally and vertically along the racks and rotated periodically to minimize potential variations in light intensity and ventilation.

The healthy control group (Ctrl) was fed a standard diet (rodent lab chow diet 5001, LabDiet, St. Louis, MO, USA), whereas the remaining groups received a high-fat diet (HFD, 60% lipids). The HFD consisted of 23.0 g casein, 18.0 g corn starch, 10.1 g sucrose, 34.0 g lard, 2.4 g vitamins, 8.0 g minerals, 3.4 g cholesterol, and 1.1 g sodium cholate [13]. After four weeks of dietary intake, diabetes was induced by intraperitoneal administration of streptozotocin (STZ). STZ 35 mg/kg was dissolved immediately before the administration in cold 0.1 M citrate buffer, pH 4.5, protected from light. To confirm the development of diabetes, blood glucose levels were measured 48 h after induction. Animals with glucose values higher than 200 mg/dL were considered diabetic and were allocated into the following groups: untreated HFD-induced animals administered vehicle (saline) (HFD, n = 5), HFD group treated with 50 mg/kg AM extract (HFD AM, n = 5), and HFD group treated with 300 mg/kg metformin (HFD Met, n = 5). AM and metformin were dissolved in distilled water. All treatments were administered orally for four weeks. The doses of AM (50 mg/kg) and metformin (300 mg/kg) were selected based on previous studies where these doses have been reported as effective in producing a hypoglycemic effect without producing adverse effects in animal models [4,7,14]. Additionally, a group of healthy, untreated animals was included as the control group, which received the vehicle (Ctrl, n = 5).

#### 2.2.5. Quantification of Biochemical Parameters

Blood samples were collected in serum separator tubes from the retro-orbital plexus of the rats before (pre-) and after (post-) treatment administration, following anesthesia with tiletamine–zolazepam (80 mg/kg, Zoletil^®^50, Virbac, Carros, France). Blood samples were centrifuged for 10 min at 1300× *g* at 4 °C, and serum was separated and stored at −70 °C until analysis. Serum glucose levels were quantified using the glucose oxidase–peroxidase method (BioSystems, Barcelona, Spain). The analyst remained blind until the final analysis of the data was completed.

To evaluate insulin resistance in the study groups, the Homeostasis Model Assessment for Insulin Resistance (HOMA-IR) was calculated both before (pre-) and after (post-) the treatment period using the following formula [15]:

HOMA-IR = [Insulin (µU/mL) × Glucose (mg/dL)]/405

Additionally, urea and creatinine levels were quantified to assess renal function, while aspartate aminotransferase (AST) and alanine aminotransferase (ALT) levels (BioSystems, Barcelona, Spain) were measured to determine hepatic function. Total cholesterol and triglycerides were also evaluated (BioSystems, Barcelona, Spain). All analytes were quantified using a BTS-350 semi-automatic analyzer (BioSystems, Barcelona, Spain).

#### 2.2.6. Quantification of Serum Insulin, Leptin, NPY, and Glucagon

Serum concentrations of insulin, leptin, NPY, and glucagon were quantified using enzyme-linked immunosorbent assay (ELISA), following the manufacturer’s instructions.. The analyst remained blind until the final analysis of the data was completed. The characteristics of each kit are described in Table 1.

#### 2.2.7. Quantification of Pancreatic Tissue Insulin and Glucagon Concentrations

At the end of the treatment period, experimental animals were euthanized by anesthetic overdose. Subsequently, the pancreas was dissected, and a tissue fragment was fixed in 4% paraformaldehyde for a minimum of 72 h. The samples were then embedded in paraffin, and 4 μm thick serial sections were prepared and mounted on charged microscope slides (Microscope slide, Premiere). Tissue sections were deparaffinized using dry heat for 10 min and rehydrated through decreasing ethanol concentrations (100%, 96%, and 80%), and antigen retrieval was performed using 10 mM citrate buffer for 10 min.

The samples were blocked with fetal bovine serum for 1 h and incubated with primary antibodies against insulin at a dilution of 1:1000 overnight at 4 °C (rat insulin, 3014, Cell Signaling Technology) or against glucagon at a dilution of 1:2000 for 1 h at 4 °C (anti-glucagon antibody, ab92517, Abcam, Cambridge, UK). Subsequently, the Mouse/Rabbit ImmunoDetector HRP/DAB Detection System kit (BSB0003, BIO SB) was used. The samples were incubated with diaminobenzidine (DAB) for 1 min, rehydrated through increasing ethanol concentrations (80%, 96%, and 100%), and counterstained with hematoxylin–eosin for 40 s. The slides were examined under a Zeiss Primo Star microscope, and photographs of all islets of Langerhans were captured using an Axiocam 208 color camera (Zeiss) with ZEISS ZEN 2.1 microscopy software (Zeiss, Baden-Württemberg, Germany). Subsequently, the percentage of insulin- and glucagon-positive areas was determined using ImajeJ 1.54i 03 software. The analyst remained blind until the final analysis of the data was completed.

#### 2.2.8. Statistical Analysis

Data were expressed as mean ± standard error of the mean (SEM). Intergroup comparisons were performed using one-way analysis of variance (ANOVA). Prior to this analysis, the assumption of homogeneity of variances was evaluated using Levene’s test. When it was satisfied (*p* > 0.05), the Bonferroni post hoc test was employed; by contrast, the Dunnett T3 (*p* < 0.05) post hoc test was utilized. Intragroup comparisons were evaluated using a two-way ANOVA with repeated measures test followed by Tukey’s post hoc test. A *p*-value < 0.05 was considered statistically significant. Statistical analyses were performed using PASW Statistics 18.0 (Chicago, IL, USA).

## 3. Results

### 3.1. Identification of Phenolic Compounds in the Methanolic Extract of AM

In research employing plant extracts, determining their composition is important as it improves experimental reproducibility and facilitates a better biological interpretation of the observed effects. Therefore, the composition of AM extract was analyzed utilizing high-performance liquid chromatography (HPLC).

**Figure 2:**
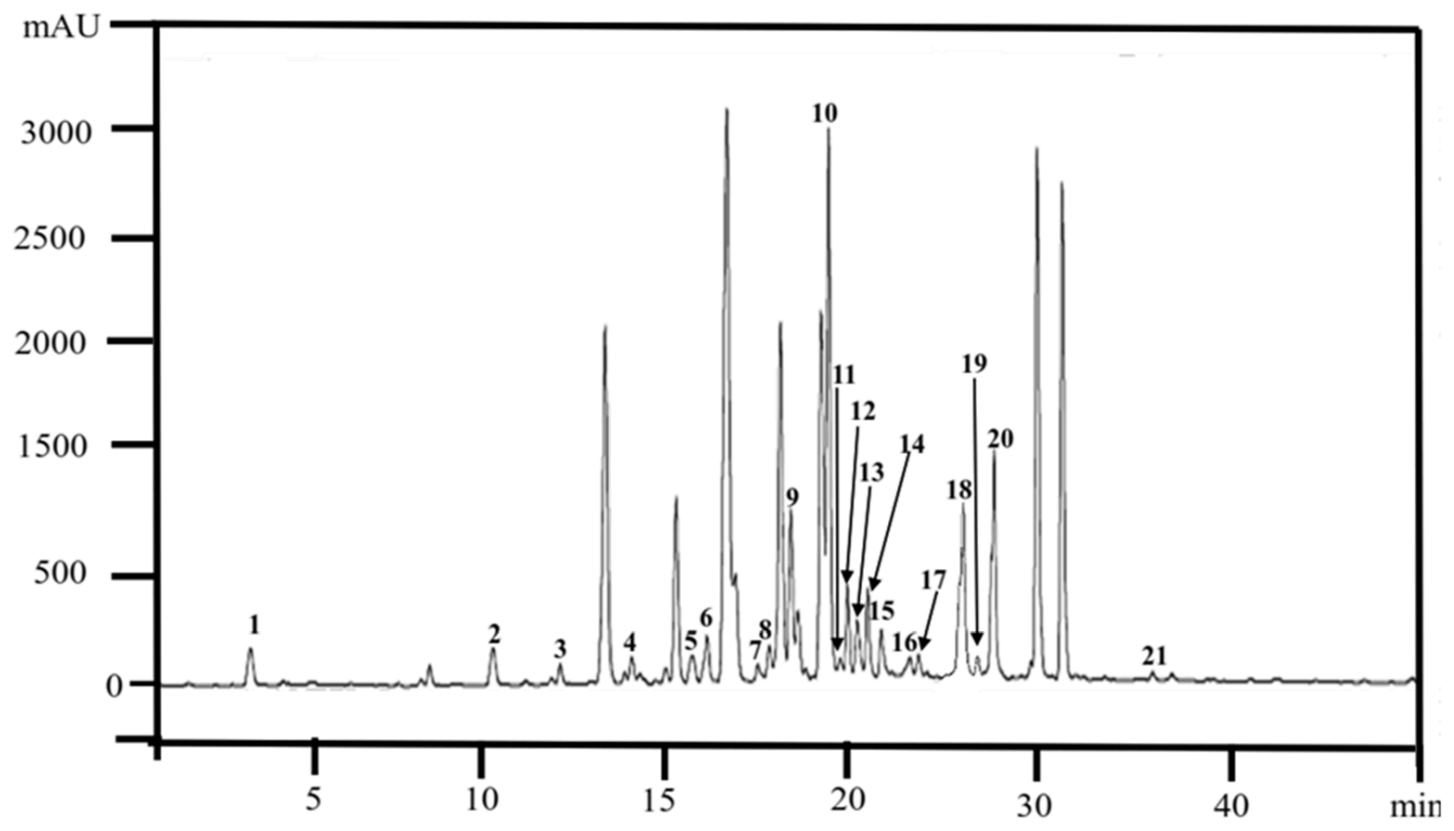
HPLC chromatogram of AM leaf methanolic extract. (1) Shikimic acid (Rt, 3.05), (2) gallic acid (Rt, 10.66), (3) protocatechuic acid (Rt, 14.82), (4) neochlorogenic acid (Rt, 17.75), (5) chlorogenic acid (Rt, 19.76), (6) 3,4-dihydroxyphenylacetic acid (Rt, 18.11), (7) 4-hydroxybenzoic acid (Rt, 18.76), (8) vanillic acid (Rt, 19.86), (9) 3-(4-hydroxyphenyl) propionic acid (Rt, 19.89), (10) epigallocatechin (Rt, 20.56), (11) caffeic acid (Rt, 21.20), (12) syringic acid (Rt, 20.57), (13) catechin (Rt, 21.90), (14) epicatechin (Rt, 22.30), (15) homovanillic acid (Rt, 22.40), (16) 4-hydroxybenzaldehyde acid (Rt, 22.76), (17) rutin (Rt, 23.41), (18) p-coumaric acid (Rt, 23.90), (19) transferulic acid (Rt, 24.23), (20) ellagic acid (Rt, 25.63) and (21) trans-cinnamic acid (Rt, 34.45). mAU: milliabsorbance units.

The extract was primarily composed of epigallocatechin, p-coumaric acid, 4-hydroxyphenylacetic acid, gallic acid, ellagic acid, epicatechin, and shikimic acid, among other substances (Figure 2 and Table 2).

**Table 2:** Phenolic chemical composition of the AM extract (mg/100 g sample).

Phenolic Compounds	mg/100 g Sample	Phenolic Compounds	mg/100 g Sample
3,4-dihydroxyphenylacetic acid	1.40 ± 0.04	Homovanillic acid	13.84 ± 0.06
4-Hydroxybenzaldehyde acid	1.47 ± 0.01	Neochlorogenic acid	5.45 ± 0.02
4-Hydroxybenzoic acid	2.56 ± 0.04	p-Coumaric acid	26.18 ± 0.02
3-(4-hydroxyphenyl) propionic acid	23.04 ± 0.51	Protocatechuic acid	0.82 ± 0.06
Caffeic acid	1.49 ± 0.01	Rutin	4.16 ± 0.09
Catechin	14.06 ± 0.17	Shikimic acid	15.45 ± 0.47
Chlorogenic acid	3.19 ± 0.01	Syringic acid	11.56 ± 0.07
Ellagic acid	16.16 ± 0.01	Trans-cinnamic acid	0.63 ± 0.01
Epicatechin	15.98 ± 0.02	Trans-ferulic acid	12.34 ± 0.04
Epigallocatechin	160.92 ± 9.97	Vanillic acid	4.10 ± 0.02
Gallic acid	18.27 ± 0.29		

### 3.2. Reduction in Serum Glucose Concentration After AM Treatment

To evaluate the effect of the AM extract on glucose levels, it was quantified before and after the treatment period. All diabetes-induced groups showed higher serum glucose levels than the untreated healthy group (Ctrl). Baseline glucose values in the HFD groups were similar (Table 3). The treated groups, HFD AM and HFD Met showed a decrease in serum glucose levels of 216.7 mg/dL (41.2%) (*p* < 0.01) and 142.0 mg/dL (25.0%), respectively, at the end of the treatment period relative to their pre-treatment values. Nonetheless, no significant difference was observed between them.

Baseline HOMA-IR values in the induced groups were higher than those in the healthy group (Ctrl; *p* < 0.05). Nevertheless, after the treatment period, reductions were observed in both the HFD Met and HFD AM groups (*p* < 0.05) compared with their respective pretreatment values (Table 3). Furthermore, the post-treatment HOMA-IR of the HFD group was higher than that of the Ctrl group (*p* < 0.05). By contrast, in the HFD AM group the post-treatment HOMA-IR values were lower (*p* < 0.05) than those of the HFD group and similar to those of the Ctrl group.

### 3.3. Effect of AM Extract on Biochemical Parameters

Total cholesterol and triglyceride levels were quantified in the study groups and remained unchanged during the experimental period in the healthy control group (Ctrl) compared with the HDF groups. The treatments reduced the cholesterol levels relative to their own baseline, the HFD AM group (38.85%, *p* < 0.05) showed a higher reduction than the metformin group (24.29%). No significant changes were observed when comparing these groups with the HFD group. Likewise, a reduction in serum triglyceride concentration relative to baseline was shown in the HFD AM (69.6%, *p* < 0.05) and HFD Met (78%, *p* < 0.05) groups (Table 3). No significant changes were observed in the comparisons across these groups; however, a comparison with the HFD group shows a reduction of 51.4% (*p* < 0.05) in HFD AM and 49.4% (*p* < 0.05) in HFD Met.

Furthermore, to evaluate renal function, serum urea and creatinine levels were quantified in the study groups. At the end of the experimental period, the treated groups exhibited reduced creatinine concentrations when compared with the HFD group, 18% HFD AM and 37% HFD Met (*p* < 0.05). The urea concentration in the HFD AM group was 23% and 37.6% (*p* < 0.05) lower compared with the HFD and HFD Met groups, respectively.

ALT and AST levels were quantified to evaluate hepatic function. AST levels were higher by 124.5% in the HFD group, 75.1% in the HFD AM group, and 20.3% in the HFD Met group, relative to the healthy group (Ctrl). A high concentration of ALT was observed in comparison to the Ctrl group of 547.5% (*p* < 0.05), 485.9% (*p* < 0.05), and 259.8% (*p* < 0.05) in the HFD, HFD AM, and HFD Met groups, respectively. It should be noted that this increase was lower in treated groups (HFD AM and HFD Met) with respect to the HFD group (Table 3).

### 3.4. Serum Insulin, Leptin, NPY, and Glucagon Concentrations

Using the ELISA method, serum concentrations of insulin, NPY, glucagon, and leptin were quantified in the study groups before and after the treatment period.

**Figure 3:**
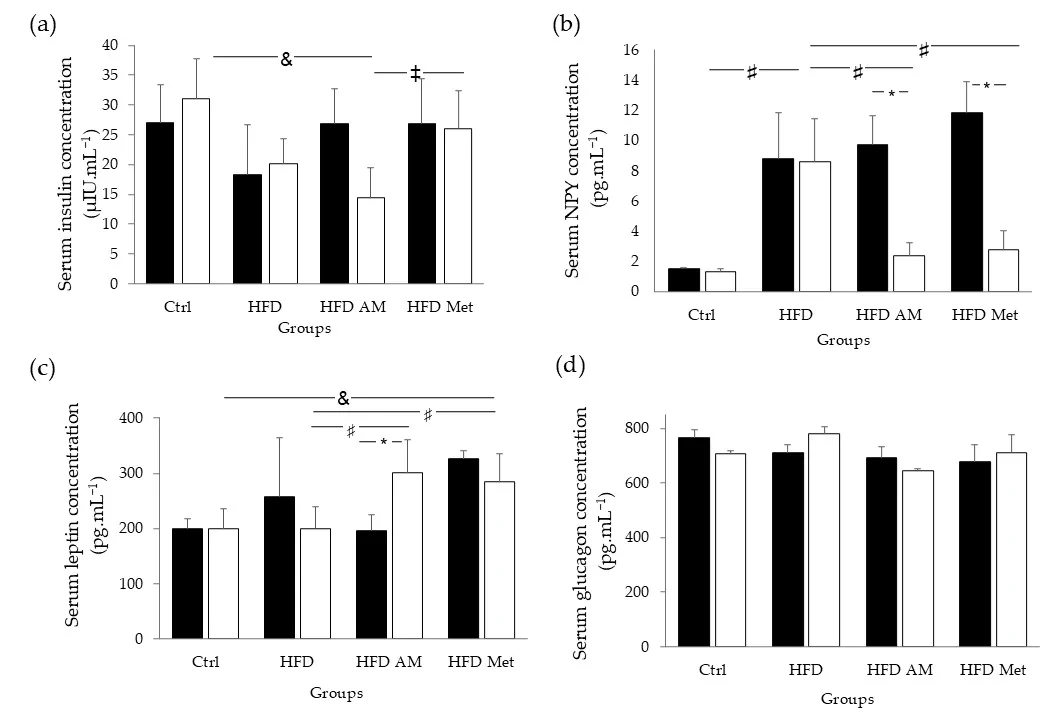
Panel (**a**): Serum insulin (µIU mL^−1^); panel (**b**): NPY (pg mL^−1^); panel (**c**): leptin (pg mL^−1^); and panel (**d**) glucagon (pg mL^−1^) concentrations. Data are expressed as mean ± SEM (n = 5 per group). Statistical significance was determined using a paired two-way ANOVA with repeated measures test followed by Tukey’s post hoc test. * *p* ˂ 0.05 comparing post- vs. pre-treatment levels. The statistical significance of the comparisons between groups was determined using a one-way ANOVA, followed by the corresponding post hoc test. ♯ *p* < 0.05 comparing respect to HFD group. ‡ comparing HFD AM with HFD Met group, and & *p* < 0.05 comparison with Ctrl group. Ctrl, control group, healthy rats without treatment; HFD, diabetic rats administered vehicle (saline); HFD AM, diabetic rats treated with AM; HFD Met, diabetic rats treated with metformin; Pre (black bar); before treatment; Post (white bar); after four weeks of treatment.

At the end of the treatment period, serum insulin concentrations were comparable to baseline values in all groups, with the exception of the HFD AM group, which exhibited a 45.9% decrease in insulin levels relative to baseline, and reductions of 28.0% (*p* < 0.05) and 44.2% (*p* < 0.05) when compared with the HFD and HFD Met groups, respectively (Table 3, Figure 3a). No differences were seen between the HFD and HFD Met groups.

The Ctrl group showed reduced pre-treatment NPY concentrations compared with the diabetes-induced groups (*p* < 0.05). In the HFD AM and HFD Met groups, a statistically significant decrease in NPY concentration (*p* < 0.05) was identified at the end of the treatment period, with reductions of 75.36% and 76.43%, respectively, compared with pre-treatment levels. In contrast, no alterations were noted in the HFD group (Figure 3b). The Ctrl group exhibited no alterations in leptin levels. While the HFD AM group demonstrated a 53.68% (*p* < 0.05) elevation in leptin levels compared with their baseline values. Leptin concentrations decreased in the HFD (22.72%) and HFD Met (13.05%) groups compared with their respective baseline values. The analysis between groups indicated that leptin concentrations were similar between the HFD AM and HFD Met groups, and significantly higher (*p* < 0.05) in contrast to the HFD group (Figure 3c). No alterations in serum glucagon levels were detected in any of the study groups when comparing pre- and post-treatment readings (Figure 3d).

### 3.5. Increased Pancreatic Tissue Insulin and Glucagon Concentrations

**Figure 4:**
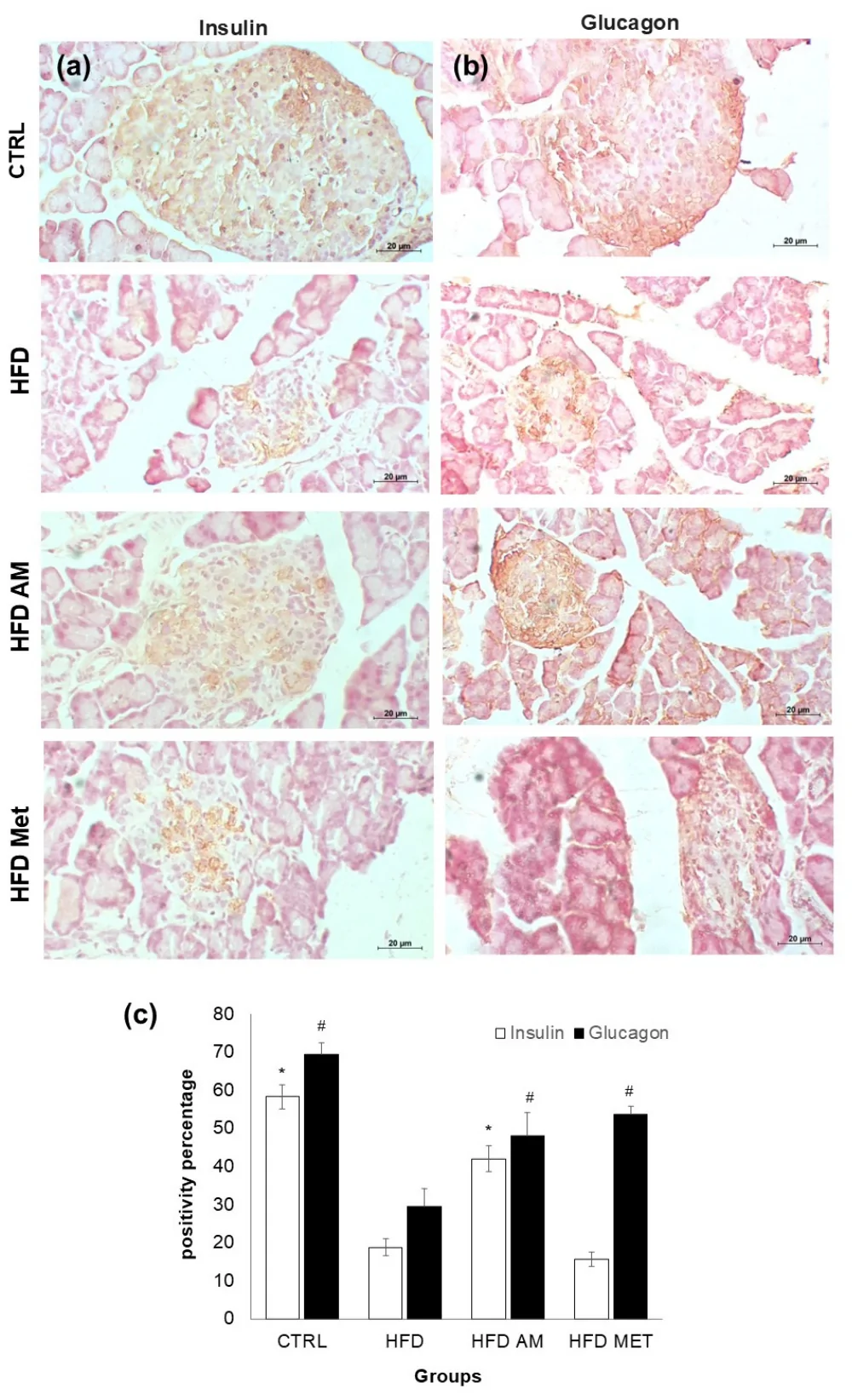
Representative microphotographs of pancreatic tissue immunostained for insulin (**a**) and glucagon (**b**) (40×). Ctrl (n = 5), control group, healthy rats without treatment; HFD (n = 5), diabetic rats administered vehicle (saline); HFD AM (n = 5), diabetic rats treated with AM; HFD Met (n = 5), diabetic rats treated with metformin. (**c**) Histogram quantification of insulin and glucagon immunopositivity (%). Data are expressed as mean ± SEM (n = 5 per group). Statistical significance was determined using ANOVA followed by post hoc. * *p* ˂ 0.05 percentage of insulin and (#) *p* ˂ 0.05 percentage of glucagon immunopositivity compared with the HFD group.

At the end of the experimental period (four weeks), pancreatic tissue samples were collected to quantify insulin and glucagon content via immunohistochemistry. The control group showed higher tissue content for both insulin (58%, *p* <0.05) and glucagon (70%, *p* <0.05) compared with all diabetes-induced groups (HFD). Interestingly, the diabetic group treated with AM (HFD AM) exhibited higher pancreatic insulin content (42%, *p* < 0.05) compared with the HFD (19%, *p* < 0.05) and HFD Met (16%, *p* <0.05) groups. Moreover, the treated groups, HFD AM and HFD Met, showed a higher percentage of glucagon-positive area (*p* < 0.05), 48% and 54%, respectively, compared with the untreated diabetic group (30%, HFD) (Figure 4). When comparing the treatment groups, a difference in insulin content is observed. However, no significant change is evident in glucagon content between these groups.

## 4. Discussion

AM, commonly known as soursop, has attracted significant interest due to its numerous biological effects, especially its antioxidant, anti-inflammatory, and hypoglycemic properties [7,16]. Phytochemical analysis revealed that this extract is rich in phenolic compounds, predominantly epigallocatechin, along with phenolic acids, catechins, and flavonoids. These compounds are known to modulate lipid homeostasis and glucose via anti-inflammatory mechanisms and to regulate insulin signaling pathways [17]. The phenolic profile of the AM extract provides a basis for the metabolic improvements observed in the present study. Flavonoids have been reported to exert hypoglycemic effects [13,14]; therefore, this finding may be attributable to the flavonoids present in the AM extract. Epigallocatechin is among the flavonoids identified in higher amounts in the AM extract (160.9 mg/100 g sample) and it has been reported to reduce glucose levels and insulin resistance in streptozotocin (STZ)-induced diabetic rats and to decrease hepatic gluconeogenesis [17,18,19]. In addition, p-coumaric acid (26.2 mg/100 g) enhances leptin signaling in Swiss albino mice [20] and gallic acid improves insulin sensitivity and protects pancreatic β-cells [21,22].

The administered dose of AM (50 mg/kg) was selected from previous research that evaluated doses of 50 and 100 mg/kg and which demonstrated that the lower dose resulted in a reduction of glucose concentration and improved other parameters, including the expression of IRS-1 and Glut 2, in a manner comparable to, or in some cases superior to, the 100 mg/kg dose [4]. Furthermore, we confirmed these findings in a pilot study where we evaluated the same doses and observed better results with 50 mg/kg of AM; therefore, this dosage was selected for testing.

Regarding the hypoglycemic effect, in our group treated with AM, a 41.1% reduction in serum glucose concentration was observed at the end of the treatment period. A study reported a 75% decrease in blood glucose levels after orally administering 100 mg/kg of AM aqueous extract for 28 days in STZ-induced diabetic Wistar rats [4]. Although the percentage reduction was lower than that reported, it is noteworthy that the dose used (50 mg/kg) in the present study was half of that employed in the aforementioned study [4], indicating that a hypoglycemic effect can also be achieved at lower doses.

Regarding lipid concentrations, metformin has been reported to improve total cholesterol and triglyceride levels [23], which is consistent with the reductions observed in these parameters after the treatment period in the HFD Met group. Similarly, a reduction in serum lipid levels was observed in the AM-treated (HFD AM) group. In this regard, this group showed a 38.85% reduction in total serum cholesterol concentration, which is higher than that reported by Sasso et al. (4.92%) after administering the same dose used in our study of an aqueous AM extract to C57BL/6 mice [5]. Furthermore, the study revealed a significant reduction in triglyceride levels only at the high doses (100 and 150 mg/kg), while no significant changes were noted at the lower dose (50 mg/kg) [5]. This contrasts with our study, where a significant reduction in triglyceride levels was observed at 50 mg/kg of AM, both in comparison with the positive control group and relative to its own baseline measurement. This comparison allows the evaluation of analyte behavior (total cholesterol and triglycerides) within the same individual after administration of the compound under evaluation, in this case, AM. The reduction in cholesterol and triglyceride levels could be associated with their polyphenol content. These compounds activate the AMPK-dependent pathways, which contributes to the regulation of lipid metabolism [24].

The reduction in serum insulin levels observed in the HFD AM group at the end of the treatment period are consistent with those reported by Son et al. [6], who administered a 50 mg/kg of AM extract to male C57BL/6 mice observing a decrease in plasma insulin concentration compared with the diabetic group [6]. These findings could be explained by the increase in post-treatment leptin levels shown in the HFD AM group (*p* < 0.05). In this regard, leptin inhibits insulin secretion in pancreatic β-cells, suppresses insulin gene expression, and improves insulin sensitivity to improve lipid profiles, including reductions in triglyceride and total cholesterol levels [25,26,27]; findings that were observed in the HFD AM group. The leptin increment in the HFD AM group could contribute to improved insulin sensitivity, as evidenced by the reduction in HOMA-IR levels (*p* < 0.05). This is consistent with a study in which 100 mg/kg was administered to diabetic male Wistar rats for 30 days, reporting a reduction in HOMA-IR compared with the diabetic group. Although only half the dose used in the study was employed in our work (50 mg/kg), a reduction in HOMA-IR was still observed [28]. The findings in the HFD AM group could explain the decrease in serum insulin levels observed, as improved insulin sensitivity reduces insulin requirements, as reflected by lower serum insulin levels at the end of the treatment period. Although the decrease in serum insulin concentration was not statistically significant after AM treatment, the significant results obtained in the HOMA-IR index integrate glucose and insulin concentrations, constituting a more sensitive indicator of insulin resistance than the individual determination of insulin [29,30].

Furthermore, NPY concentration was quantified. NPY is an orexigenic neuropeptide that regulates food intake by inducing the sensation of hunger [31]. It is considered a diabetogenic neuropeptide that increases levels in diabetes; however, its evaluation in diabetic conditions is relevant [32]. This correlates with the higher baseline levels observed in the diabetes-induced groups compared with the control group. However, after treatment administration (HFD AM and HFD Met), a statistically significant reduction in serum NPY levels was observed. It has been reported that after metformin was administered to obese diabetic rats orally, their food intake decreased, their glucose control improved, and NPY and AgRP expression in the hypothalamus decreased [33]. Furthermore, leptin can reduce NPY levels [34], consistent with the findings for these parameters in the HFD AM group.

Pancreatic insulin tissue content was significantly lower in the HFD group. In type 2 diabetes models, there is a 25% to 60% reduction in the number and function of pancreatic β-cells, resulting from β-cell dysfunction and apoptosis, leading to reduced insulin production [35]. Despite a decrease compared with the control group, the HFD AM group showed a significantly higher pancreatic insulin content than the HFD group (*p* < 0.01), indicating partial tissue insulin recovery and cytoprotective effect on β-cells. Hyperglycemia and hyperlipidemia have been reported to increase free radical production, thereby elevating oxidative stress and impairing β-cell viability. Thus, antioxidants may improve pancreatic function by reducing inflammation and cellular damage. In particular, AM has been shown to possess antioxidant activity attributed to its polyphenol content, which may contribute both to the preservation of islet structure and to improvements in insulin synthesis and regulation [36,37]. As mentioned, improved insulin sensitivity reduces insulin secretion, in turn increasing pancreatic insulin content [34], consistent with the findings in the HFD AM group.

Similar serum glucagon concentrations were observed across all experimental groups. However, a slight increase was detected in the HFD group compared with the control and HFD AM groups. This finding may be explained by the fact that experimental animals in both the healthy (Ctrl) and diabetic groups (HFD, HFD AM, and HFD Met) were fasted, which stimulates glucagon secretion. This occurs because, although these animals exhibit hyperglycemia, they are in a state of cellular fasting [38]. Therefore, in future studies, glucagon levels could be quantified at multiple time points, including the postprandial state.

Although no significant changes in serum glucagon concentration were observed, significant differences in pancreatic tissue glucagon content were detected among the study groups, indicating that the observed effects were tissue-specific rather than systemic. In this regard, the control (Ctrl) group showed higher tissue glucagon content compared with the diabetes-induced groups (HFD, HFD AM, and HFD Met). This is expected, as pancreatic α-cells secrete glucagon in a regulated manner during fasting conditions [39]. By contrast, in type 2 diabetes, pancreatic α-cell function is impaired, leading to dysregulated and increased glucagon secretion; consequently, this is associated with lower tissue glucagon content in the islets of Langerhans, as observed in the present study, where the HFD group exhibited lower pancreatic glucagon content compared with the rest of the groups [40]. The groups treated with AM and metformin exhibited a significantly higher pancreatic glucagon content (*p* < 0.01) compared with the HFD group; this increase was slightly higher in the HFD Met group. In this regard, in streptozotocin-induced diabetic rats, metformin stimulated partial pancreatic islet regeneration and significantly decreased plasma glucagon levels, which could have contributed to the drug’s antihyperglycemic activity via lowering hepatic gluconeogenesis [41]. The findings indicate that AM extract may influence pancreatic α-cells, possibly by partially restoring the pancreatic microenvironment or energy homeostasis; more studies are necessary to verify this hypothesis.

In addition, it is important to corroborate the safety of the AM extract on liver and kidney functions. For this purpose, AST and ALT concentrations (enzymes used to assess hepatic function) and urea and creatinine levels (which evaluate renal function) were quantified [42]. It has been reported that STZ produces liver dysfunction, increasing AST and ALT levels [43]. This is consistent with our results, in which diabetes-induced groups showed increased serum AST and ALT concentrations compared with the Ctrl group at the end of the experimental period. However, the treated groups (HFD AM and HFD Met) exhibited lower levels of AST and ALT post-treatment in comparison with the HFD group. It has been reported that metformin reduces ALT and AST levels and ameliorates histological changes, partially mitigating liver damage [44]. A study in which male rats received 100, 200, and 300 mg/kg of an ethanolic extract of AM leaves for 30 days. Only the highest dose (300 mg/kg) increased AST, ALT, and ALP concentrations. Therefore, the authors recommended the use of lower doses of the extract, with which no hepatic damage was observed [42]. In our study, a lower dose (50 mg/kg) than that tested by Nweke et al. [42] was used. Furthermore, the increase in hepatic enzyme levels observed in the HFD AM group at the end of treatment was lower than that observed in the HFD group. Thus, it can be inferred that these changes are attributable to diabetes rather than to extract administration. It could even be speculated that AM may exert a protective effect against hepatic damage induced by the experimental model; however, further studies are required to confirm this hypothesis. On the other hand, our research only evaluated the liver function through these enzymes; however, histological analysis of the liver is required to evaluate any structural improvements after treatment with AM.

The creatinine concentration after the experimental period in the Ctrl group and the treatment groups (HFD AM and HFD Met) remained within normal values (0.6–0.9 mg/dL) [45,46,47]. In the Ctrl and HFD AM groups, the urea concentration showed normal values (34.5–50.16 mg/dL). It has been reported that STZ administration increases urea and creatinine concentrations as time passes [48]. Therefore, the slight increase in creatinine in the HFD group and urea levels in the HFD and HFD Met groups may be indicative of initial damage to kidney function caused by the experimental group. It should be noted that there was no increase in these parameters in the group treated with AM. In this respect, a study of male albino rats administered 7,12-dimethylbenz[a]anthracene and a 200 mg/kg dose of aqueous AM extract showed lower urea and creatinine concentrations than the untreated damage group [49]. Our findings suggest that the AM extract administered at the specified dose did not adversely affect renal function. However, it is necessary to perform histological analyses to discard detrimental effects at the structural level.

In summary, AM treatment at a dose of 50 mg/kg showed significant improvements in glucose, triglycerides, NPY, leptin serum concentrations, and glucagon tissue content, similar to that observed in the HFD Met group. However, it is worth noting that the effect on HOMA-IR, urea and insulin pancreatic content was significantly different to the reference drug (HFD Met). Therefore, these findings support the therapeutic potential of AM and suggest that its beneficial effect may be mediated through mechanisms similar to metformin, as well as through additional pathways that could complement its therapeutic actions. Future studies are required to verify this hypothesis.

Finally, the study provides insights into the effect that AM has on glucagon content in pancreatic tissue in a T2DM model; recently, glucagon has gained interest as a key molecule in the management of T2DM. Additionally, it explores the effect of AM in appetite regulation and energy balance through leptin and NPY serum levels, key molecules that are deregulated in T2DM. This research establishes a basis for studying the possible therapeutic effects of AM in pathways associated with energy balance.

## 5. Conclusions

The results of the present study demonstrate the potential therapeutic effect of the methanolic extract of AM in a murine model of streptozotocin- and high-fat-diet-induced diabetes. The findings reveal that oral administration of 50 mg/kg of the extract for four weeks partially modulates pancreatic tissue expression of insulin and glucagon and improves metabolic regulation through modulation of NPY and leptin. These results support previous evidence regarding the beneficial effects of AM in glucose metabolism and provide additional insight into the mechanisms that may underline its antidiabetic action. Therefore, AM may represent a potential complementary therapeutic agent in the treatment of T2DM; however, further studies are required to fully elucidate its mechanism of action on the signaling pathways of molecules involved in T2DM.

## 6. Limitations

Serum evaluations were performed in the fasting state. However, the fasting state influences serum glucagon levels, which explains why similar results were obtained in all study groups, both pre- and post-treatment, making it difficult to observe the effect of the treatments on this hormone. Unfortunately, due to the nature of the study, this could not be evaluated at other times. Therefore, in future studies, the serum concentration of glucagon could be evaluated in postprandial conditions in order to be able to observe the effect of AM on circulating glucagon levels. However, it was possible to evaluate the effect of treatments on the glucagon content in pancreatic tissue. This is because the glucagon content in pancreatic tissue is regulated over a longer period, reflecting a chronic adaptation that makes it less susceptible to changes induced by fasting. Another limitation of the study is that only kidney and liver functions were evaluated through the quantification of serum biomarkers; however, it would be important to perform a histological evaluation to evaluate damage at a structural level in these organs.

Moreover, the extraction procedure employed results in a polyphenol-rich extract; thus, characterization mainly focused on quantifying their concentration, but the presence of other phytochemical constituents could also be examined.

## Figures and Tables

**Table 1:** Insulin, leptin, NPY and glucagon ELISA kit characteristics.

Kit	Sample Amount	Minimum Detectable Concentration
DRG Insulin Rat ELISA, catalog EIA-2048, DGR Instruments GmbH, Marburg, Germany	100 µL	5 µIU/mL
Rat Leptin ELISA Kit, catalog ab229891, Abcam, Cambrige, UK	50 µL	24 pg/mL
Rat Neuropeptide Y ELISA Kit, catalog MBS700432, MyBioSource, San Diego, CA, USA	100 µL	0.39 pg/mL
Glucagon quantikine ELISA Kit, catalog DGCG0, R&D Systems, Minneapolis, MN, USA	50 µL	1.12 g/mL

**Table 3:** Pre- and post-treatment levels of serum biochemical parameters.

Groups		Ctrl	HFD	HFD AM	HFD Met
Glucose (mg/dL)	Pre	133.2 ± 12.0	509.3 ± 17.3	525.8 ± 23.0	425.0 ± 28.7
	Post	99.6 ± 11.9	441.3 ± 39.6	309.2 ± 47.5 *	318.5 ± 84.0
Insulin (µUI/mL)	Pre	26.9 ± 6.5	18.4 ± 8.3	26.8 ± 5.9	26.8 ± 7.6
	Post	31.0 ± 6.8	20.1 ± 4.2	14.5 ± 4.9	25.9 ± 6.5
HOMA-IR Index	Pre	6.7 ± 2.2	21.6 ± 3.7	29.3 ± 7.7	29.5 ± 11.1
	Post	8.0 ± 1.9 ^†^	21.8 ± 4.9	6.7 ± 1.2 *^†^	18.5 ± 7.5
Total cholesterol (mg/dL)	Pre	41.0 ± 5.5	209.7 ± 30.1	314.5 ± 45.6	378.7 ± 106.2
	Post	54.6 ± 2.7	169.0 ± 68.9	192.3 ± 28.9 *	286.7 ± 31.6 ^‡^
Triglycerides (mg/dL)	Pre	44.0 ± 3.1	250.8± 53.4	269.8 ± 46.4	386.7 ± 71.1
	Post	42.0 ± 5.5	168.8 ± 23.7	82.0 ± 17.2 *^†^	85.3 ± 4.4 *^†^
Creatinine (mg/dL)	Pre	0.70 ± 0.01	0.7 ± 0.02	0.67 ± 0.02	0.70 ± 0.04
	Post	0.80 ± 0.06	1.0 ± 0.12	0.82 ± 0.05	0.63 ± 0.03 ^†^
Urea (mg/dL)	Pre	46.7 ± 6.0	35.4 ± 2.4	37.3 ± 2.1	43.3 ± 3.8
	Post	47.3 ± 5.8	50.8 ± 5.6	39.1 ± 4.0 ^‡^	62.7 ± 3.5 ^‡^
AST (U/L)	Pre	164.0 ± 3.1	168.3 ± 10.2	177.2 ± 28.2	221.3 ± 5.0
	Post	187.3 ± 27.0	420.5 ± 80.9 *	328.0 ± 46.1	225.3 ± 27.5
ALT (U/L)	Pre	54.7 ± 5.0	86.7 ± 25.3	77.4 ± 10.0	118.0 ± 24.3
	Post	50.3 ± 2.2	325.7 ± 53.2 *	244.4 ± 43.2 **	181.0 ± 50.0

Data are expressed as mean ± SEM (n = 5 per group). Statistical significance of the comparisons between pre- and post-treatment measurements was determined using a paired two-way ANOVA with repeated measures test followed by Tukey’s post hoc test. ** *p* ˂ 0.01, * *p* ˂ 0.05. The statistical significance of the comparisons between groups was determined using a one-way ANOVA, followed by the corresponding post hoc test. † *p* < 0.05 comparison respect to HFD group. ‡ *p* < 0.05 comparing the HFD AM with the HFD Met group. ALT, alanine aminotransferase; AST, aspartate aminotransferase; Ctrl, control group, healthy rats without treatment; HFD, diabetic rats administered vehicle (saline); HFD AM, diabetic rats treated with AM; HFD Met, diabetic rats treated with metformin; Pre, before treatment; Post, after four weeks of treatment.

## Data Availability

The datasets are available upon request from the corresponding author.

## Supplementary Materials

The following supporting information can be downloaded at: https://www.mdpi.com/article/10.3390/life16081219/s1, Table S1: Characteristics of the standards used in HPLC.

## Abbreviations

The following abbreviations are used in this manuscript:

AKTp	Phosphorylated AKT
ALT	Alanine aminotransferase
AM	Annona muricata
AMPKp	AMP-activated protein kinase
ANOVA	Analysis of variance
AST	Aspartate aminotransferase
C/EBPα	CCAAT/enhancer-binding protein alpha
Ctrl	Control
T2DM	Type 2 diabetes
ELISA	Enzyme-linked immunosorbent assay
FAS	Fatty acid synthase
GLUT2	Glucose transporter 2
HbA1c	Glycated hemoglobin
HFD	High fat diet
HFD AM	High fat diet with *A. muricata*
HFD Me	t High fat diet with metformin
HOMA-IR	Homeostasis Model Assessment for Insulin Resistance
HPLC	High-performance liquid chromatography
Met	Metformin
NPY	Neuropeptide Y
PPARγ	Peroxisome proliferator-activated receptor gamma
SEM	Standard error of the mean
SREBP1c	Sterol regulatory element-binding protein 1c
STZ	Streptozotocin
