# Supplementary material for: Annona muricata L. Leaf Methanolic Extract Modulates Glucose Homeostasis and Improves Pancreatic Hormones Protein Expression in Diabetic Rats
Source: Life (Basel). 2026 Jul 23;16(8):1219. doi: 10.3390/life16081219 (PMC13514405; doi:10.3390/life16081219)
Supplement: Supplementary file 1 [file life-16-01219-s001.zip › life-4425539-supplementary.pdf]

Table S1. Characteristics of the standards used in HPLC

Phenolic compounds	Purity	Concentration (µg/ml)	R-squared	Formula	Detection limit (LOD, µg/ml)
3,4-dihydroxyphenylacetic acid	98%	3.13-50	0.9987	y=117.31x-91.121	0.11
4-hydroxybenzaldehyde acid	98%	3.13-50	0.9966	y=1083.8x+415.7	0.01
4-hydroxybenzoic acid	≥99%	3.13-50	0.9989	y=376.93x-287.21	0.08
3-(4-hydroxyphenyl) propionic acid	98%	6.25-100	0.9991	y=47.712x-21.511	0.32
Caffeic acid	≥98%	3.13-50	0.9995	y=637.32x-421.92	0.03
Catechin	≥97%	3.13-50	0.9992	y=84.567x-63.346	0.22
Chlorogenic acid	≥95%	6.25-100	0.9992	y=389.28x-533.98	0.05
Ellagic acid	≥95%	3.13-50	0.9986	y=591.73x+411.55	0.02
Epicatechin	≥98%	6.25-100	0.9997	y=133.9x-130.53	0.20
Epigallocatechin	≥95%	6.25-100	0.9995	y=23.341x-31.289	0.55
Gallic acid	≥99%	6.25-100	0.9979	y=262.9x-402.91	0.07
Homovanillic acid	>99%	3.13-50	0.9997	y=90.436x-42.411	0.34
Neochlorogenic acid	≥98%	12.5-200	0.9984	y=288.88x-947.31	0.08
p-coumaric acid	≥98%	6.25-100	0.9695	Y=496.93x+1844.1	0.04
Protocatechuic acid	≥99%	3.13-50	0.9997	y=391.51x-112.28	0.05
Rutin	≥94%	3.13-50	0.9998	y=137.5x-51.767	0.17
Shikimic acid	≥99%	156.25-2500	0.9967	y= 1.3354x-2.52	0.49
Syringic acid	≥95%	6.25-100	0.9993	y=395.91x-320.34	0.07
Trans-ferulic acid	99%	3.13-50	0.9984	y= 343.49x-328.28	0.09
Trans-cinnamic acid	≥99%	3.13-50	0.9983	y=1070.9x-1271.9	0.02
Vanillic acid	≥97%	6.25-100	0.9997	y=286.35x-300.01	0.06
